# Twelfth Annual Report of the New Jersey Board of Agriculture

**Published:** 1885-07

**Authors:** 


TWELFTH ANNUAL REPORT OF THE NEW JERSEY BOARD OF AGRICULTURE. Trenton, 1885.

This volume, besides the portion devoted to agricultural subjects, publishes addresses made by Drs. J. D. Hopkins, E. M. Hunt, W. B. E. Miller, on Diseases of Domestic Animals, and Dr. Wm. Herbert Lowe, of Paterson, on "Canine Madness in our Midst," showing that hydrophobia is on the increase in that State, and calling for legislation to have the power vested in the proper officials to destroy dogs on reasonable suspicion of having the disease.

